# Titanium Dioxide Nanofibers and Microparticles Containing Nickel Nanoparticles

**DOI:** 10.5402/2012/816474

**Published:** 2012-10-12

**Authors:** Faheem A. Sheikh, Javier Macossay, Muzafar A. Kanjwal, Abdalla Abdal-hay, Mudasir A. Tantry, Hern Kim

**Affiliations:** 1 Department of Chemistry, University of Texas-Pan American, Edinburg, TX 78539, USA; 2 DTU Food, Technical University of Denmark, Soltofts Plads, Building 227 2800 Kgs. Lyngby, Denmark; 3 Department of Bio and Nano System Engineering, College of Engineering, Chonbuk National University, Jeonju 561-756, Republic of Korea; 4 Department of Mechanical Design and Materials Engineering, Chonbuk National University, Jeonju 561-756, Republic of Korea; 5 National Center for Natural Products Research, Research Institute of Pharmaceutical Sciences, School of Pharmacy, The University of Mississippi, Oxford, MS 38677, USA; 6 Department of Environmental Engineering and Biotechnology, Energy & Environment Fusion Technology Center, Myongji University, Kyonggi-do, Yongin 449-728, Republic of Korea

## Abstract

The present study reports on the introduction of various nanocatalysts containing nickel (Ni) nanoparticles (NPs) embedded within TiO_2_ nanofibers and TiO_2_ microparticles. Typically, a sol-gel consisting of titanium isopropoxide and Ni NPs was prepared to produce TiO_2_ nanofibers by the electrospinning process. Similarly, TiO_2_ microparticles containing Ni were prepared using a sol-gel syntheses process. The resultant structures were studied by SEM analyses, which confirmed well-obtained nanofibers and microparticles. Further, the XRD results demonstrated the crystalline feature of both TiO_2_ and Ni in the obtained composites. Internal morphology of prepared nanofibers and microparticles containing Ni NPs was characterized by TEM, which demonstrated characteristic structures with good dispersion of Ni NPs. In addition, the prepared structures were studied as a model for hydrogen production applications. The catalytic activity of the prepared materials was studied by in situ hydrolysis of NaBH_4_, which indicated that the nanofibers containing Ni NPs can lead to produce higher amounts of hydrogen when compared to other microparticles, also reported in this paper. Overall, these results confirm the potential use of these materials in hydrogen production systems.

## 1. Introduction

In recent years, due to concerns about global warming and the depletion of fossil fuels from the natural reservoirs, the utilization of various other sources of energy had been intensively investigated by scientific society. There are various means of obtaining energy from the natural and artificial resources. Among the various forms of energy, hydrogen has become one of the most promising future energy means of harvesting. However, the production of this important source by direct water splitting without any byproducts is one of potential alternatives to hydrogen fuel for future energy supply [1, 2]. In order to overcome this rising demand for hydrogen, many methods have been devised, such as reforming of natural gas [3, 4], coal gasification [5], biomass pyrolysis and gasification [6], hydrolysis of chemical hydrides [7, 8], and electrolytic or photocatalytic water splitting [1, 2]. Although the hydrogen production by water splitting using electrolysis of alkaline solution is commercially done, the efficiency of the process is low. However, efficiencies are increased through the use of polymer electrolyte membranes and photovoltaic reactions. Recently, there has been a growing interest in hydrogen generation and storage using metal hydrides, such as lithium hydride (LiH) [9], sodium 2 ISRN Nanomaterials aluminum hydride (NaAlH_4_) [10], lithium borohydride (LiBH_4_) [11], and sodium borohydride (NaBH_4_) [12–14]. NaBH_4_ is the most favorable compound for hydrogen production because of its high hydrogen density, stability in alkaline solution [15], pure hydrogen generation [16], and recycling of the byproducts [17]. The governing reaction for hydrogen storage and generation is given as

(1) $$NaBH_{4}+2H_{2}O\to NaBO_{2}+2H_{2}\Delta H=300KJ/\text{mol}.$$

Similarly, the strategies to use the metal-catalyst in presence of NaBH_4_ for accelerating the rate of hydrogen production had recently been accomplished. These various metal-catalysts include the platinum (Pt) [18], palladium (Pd) [19], ruthenium (Ru) [20], cobalt (Co) [21], Co-B [22], nickel (Ni) [23], Ni-B [13], Ni-Co-B [24], and carbon nanotubes (CNT) [25] had been extensively utilized for the hydrogen production. Nickel metal is considered economically promising compared with other catalyst-forms especially with that of platinum. This is the reason the use of nickel-based catalysts had been evaluated for the production of hydrogen [23, 24]. It is noteworthy to mention that Ni metal in the form of nanoparticulate in pure or associated form can be effectually used to boost the production of hydrogen when we used NaBH_4_ [23, 24]. Having these essential properties, we have selected nickel nanoparticles and NaBH_4_ as model catalyst for hydrogen production for the present study.

The electrospinning technique has attracted considerable attention due to the production of fibers with diameters that range from the micrometer to the nanometer size [26, 27]. In a typical electrospinning process, an electrostatically driven polymer jet is ejected from a polymer solution or a sol-gel which undergoes a bending instability wherein the solvent evaporates, and an ultrafine stretched fiber is deposited on a grounded collector [28]. Consequently, the nanofibers obtained by this technique possess large surface areas when compared to other nanoparticle forms [29].

The present work presents the fabrication of various kinds of nanocatalysts forms and their capability to produce hydrogen. The fabricated nanoforms were fabricated as pure TiO_2_ nanofibers, modified TiO_2_ nanofibers containing Ni nanoparticles (NPs) and TiO_2_ microparticles containing Ni NPs. These prepared nanocatalysts have been intensively studied and well characterized with various states of the art techniques. After characterization, the efficiency of these materials was tested for the production of hydrogen through in situ hydrolysis of NaBH_4_.

## 2. Experimental Section

### 2.1. Materials

Poly(vinyl acetate) (PVAc, Mw = 500,000 g/mol) was obtained from Sigma Aldrich, USA. Titanium (IV) isopropoxide [Ti(Iso)], 98% assay was purchased from Junsei Co. Ltd., Japan. Nickel nanopowder < 100 nm, 99.9% pure was purchased from Aldrich, USA. *N,N*-dimethylformamide (DMF) was obtained from Showa Chemicals Ltd., Japan and used without further purification.

### 2.2. Characterization

The morphology of the obtained nanocatalysts was analyzed utilizing a JEOL JSM-5900 scanning electron microscope, JEOL Ltd., Japan. The phase and crystallinity of the nanofibers and microparticles was investigated using an X-ray diffractometer (XRD, Rigaku Co., Japan) with Cu Kα (λ = 1.540 °A) radiation over a Bragg angle ranging from 20 to 80° Transmission electron microscopy (TEM) was done with a JEOL JEM 2010 operating at 200 kV, JEOL Ltd., Japan.

### 2.3. Procedure

#### 2.3.1. Fabrication of Nanofibers by Electrospinning

**Scheme 1:**
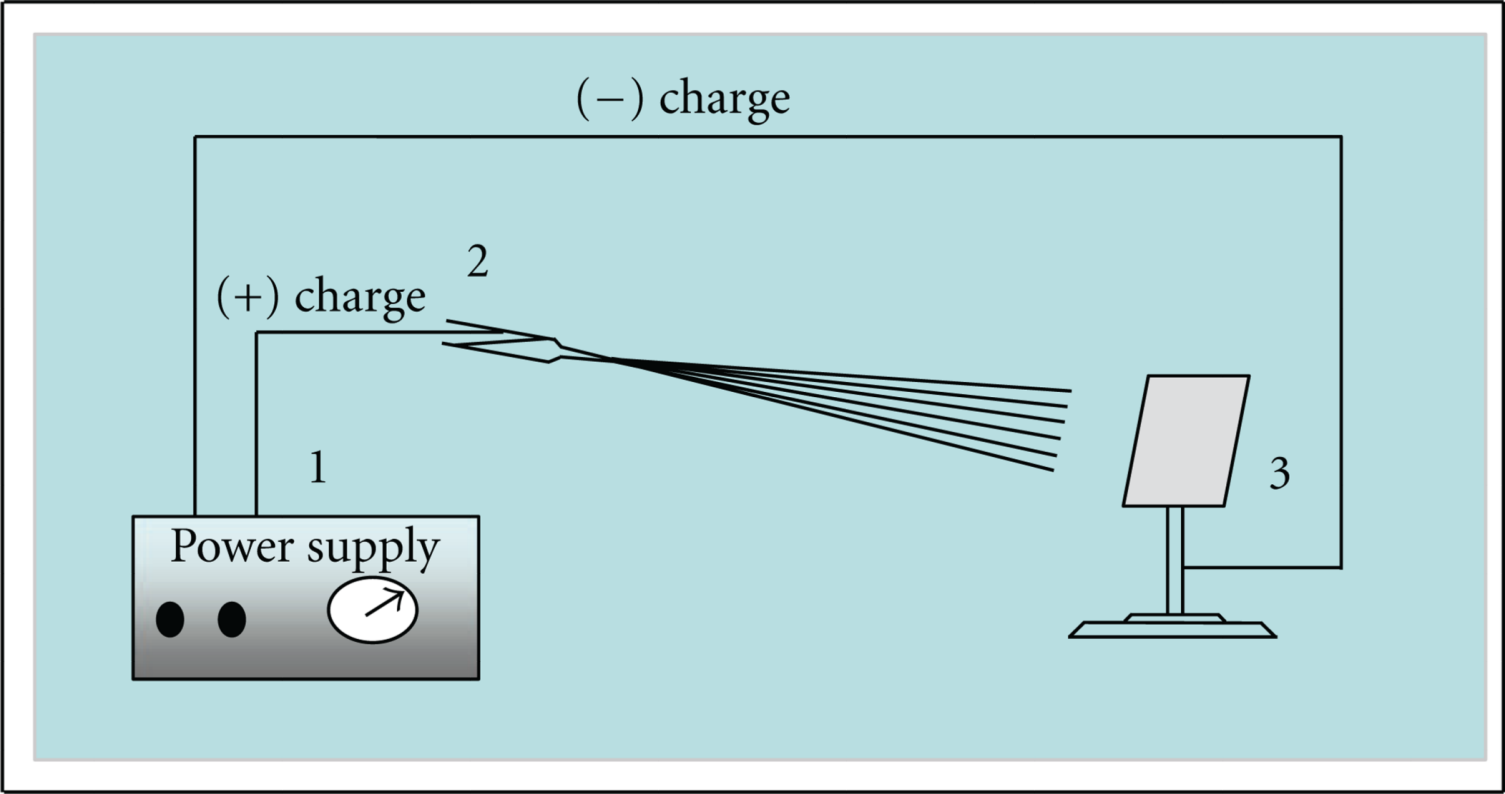
The schematic illustration of a simple electrospinning spinning apparatus: (1) dc power supply (2) syringe, and (3) collector.

The electrospinning process was utilized to produce TiO_2_ nanofibers containing Ni NPs. Typically, a sol-gel was prepared by mixing Ti(Iso) and PVAc (20wt%, in DMF) with a weight ratio of 2:3. Thereafter, a few drops of acetic acid were added until the solution became transparent under stirring. To fabricate the sol-gels containing Ni NPs, a step by step methodology was adopted. Briefly, 0.5 g of nickel NPs, were added into a previously prepared transparent solution of Ti(Iso)/PVAc; the solution was subsequently homogenized under stirring for 10 minutes. A high voltage power supply (CPS-60 K02V1, Chungpa EMT Co., Republic of Korea), capable of generating voltages up to 60 kV, was used for electrospinning the sol-gels. The solution to be electrospun was supplied through a plastic syringe attached to a capillary tip, which contained a copper pin to connect to the positive electrode (anode) in the high power supply. The electrospinning system was completed through the attachment of the negative electrode (cathode) to a grounded metallic collector. The nanofibers were deposited on rectangular collector covered with thin sheet of aluminum foil, equipped with heating system having temperature of 40°C, which helps to remove the residual solvents after the fiber lands on collector (Scheme 1). The solutions were electrospun at 15 kV and 15 cm working distance (the distance between the needle tip and the collector). The as-spun fibers were initially dried for 24 h at 80°C under vacuum in the presence of P_2_O_5_, to remove the remaining residual solvents. In order to remove the polymer used in making sol-gels, the samples were additionally heated in air atmosphere at 600°C for 1h with heating rate of 5°C/min.

#### 2.3.2. Fabrication of Microparticles by Sintering

A sol-gel was prepared by mixing Ti(Iso) and PVAc (20wt% in DMF) with a weight ratio of 2 : 3, respectively. Thereafter, a few drops of concentrated acetic acid were added under stirring to afford a transparent solution, to which 0.5 g of nickel NPs were added. Hereafter, these solutions were homogeneously mixed under stirring for 10 minutes. However, instead of electrospinning as previously described for fabrication of nanofibers, this solution was dried under vacuum at 80°C for 48 h to completely remove the solvents. The obtained solid materials were finely ground and sintered in air at 600°C for 1 h with a heating rate of 5°C/min. After the sintering process, the samples were further subjected to fine grinding to additionally reduce their size.

#### 2.3.3. Hydrogen Production Studies

All the samples were investigated for their catalytic activity for the in situ hydrolysis of NaBH_4_. Typically, 50mg of all the sample combinations were placed in a specially designed tight sealed flask which contained 50mL of a distill water containing 50 mg of NaBH_4_ at constant temperature (25°C). The catalytic performances were compared for the hydrogen production from hydrolysis of NaBH_4_. Briefly, the reaction proceeded at a stirring rate of (1000 rpm) and the amount of hydrogen generated over time was measured immediately after all the components were added through the water displacement method [30], where the volume of hydrogen is equal to that of the displaced water whose weight was recorded by a balance. The balance connected with a computer, which was installed with the software of “Balance Talk”, can read the weight of balance automatically.

## 3. Results and Discussions

**Figure 1:**
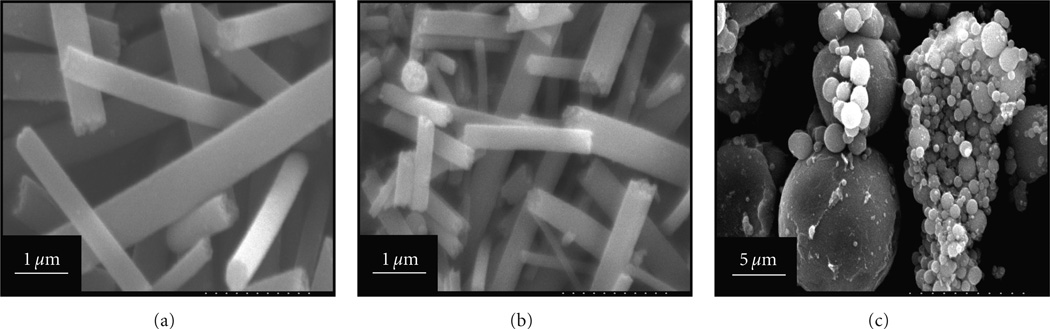
SEM images of the obtained catalysts after the calcinations process. Images of the pristine TiO_2_ nanofibers (a), images of the TiO_2_ nanofibers containing Ni NPs (b), and TiO_2_ microparticles containing Ni NPs (c). Calcination Sol-gel containing Ti(Iso) and nickel nanoparticles Electrospinning Nickel nanoparticles Nanofibers before calcination Nanofibers after calcination

In these experiments, PVAc was used as a binder for making the sol-gels to get good viscous solutions, so as to have the appropriate bending instability during the electrospinning process. After getting the nanofiber mats from electrospinning of the sol-gels, the obtained nanofiber mats were dried, and further subjected to frying in a furnace to remove the polymer binder (i.e., PVAc). In this context, (Figure 1(a)) shows the SEM images of nanofibers obtained after performing the frying process. As shown in this figure, the morphology of pristine nanofibers (after calcination) consists of pure TiO_2_ and is not affected by the high temperature heating. According to the high temperature of calcination used at 600°C, which is twice the thermal degradation temperature of PVAc [31], this temperature should have been sufficient to remove the PVAc completely, leaving behind only ceramic TiO_2_. As can be observed in this image, that nanofiber morphology is in accordance with our previously established reports [32]. Furthermore, this image allows us to determine the average diameters of obtained nanostructures by using (Photoshop 6), around (10–12 individual diameters were measured per sample). The resultant diameters in case of pure TiO_2_ nanofiber were to be 600 ± 320 nm.

**Scheme 2:**
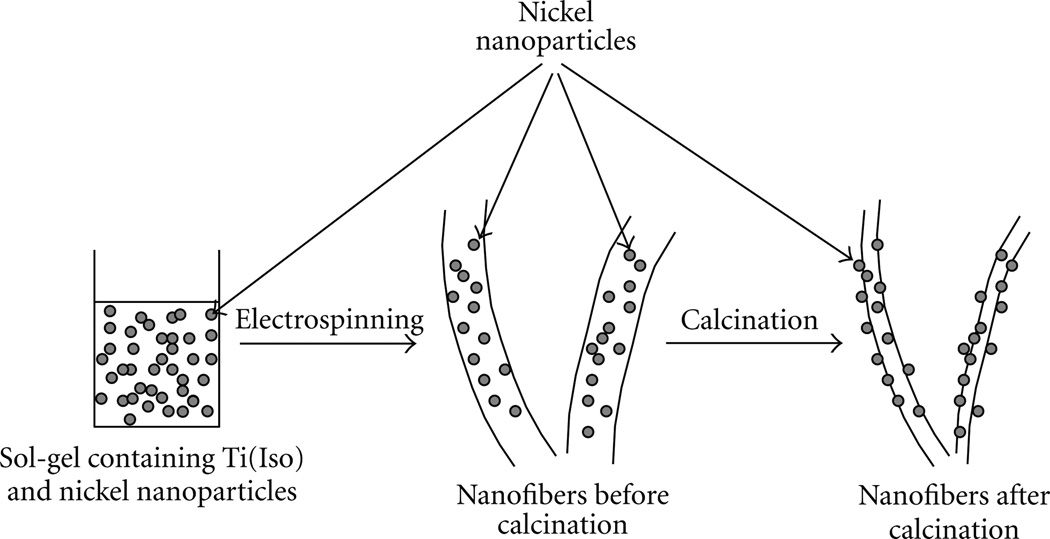
Representation of this novel strategy to fabricate TiO_2_ nanofibers containing Ni NPs.

Figure 1(b) shows the SEM morphology of the nanofibers containing Ni NPs. It is observed that the morphology of the nanofibers was not affected by the addition of the Ni NPs, if the nanofibrous morphology is considered. However, an important observation was observed that nanofibers containing Ni presented a smaller diameter than the pristine TiO_2_ nanofibers. Moreover, the average fiber diameter calculated was to be 982 ± 391 nm for nanofibers containing Ni NPs, which is comparatively less than (600 ± 320 nm) as that was seen in case of pristine TiO_2_ nanofibers. It is likely that the colloidal solutions used for electrospinning were highly conductive after addition of Ni, thus promoting a reduction in the fiber diameter [33]. A schematic representation for the fabrication of nanofibers is produced in (Scheme 2). Briefly, the first step of this scheme includes the preparation of a sol-gel containing Ni NPs. After this step, the prepared sol-gel is electrospun and calcinated to remove the binder (PVAc). The final step results in the formation of TiO_2_ nanofibers containing Ni NPs.

**Figure 2:**
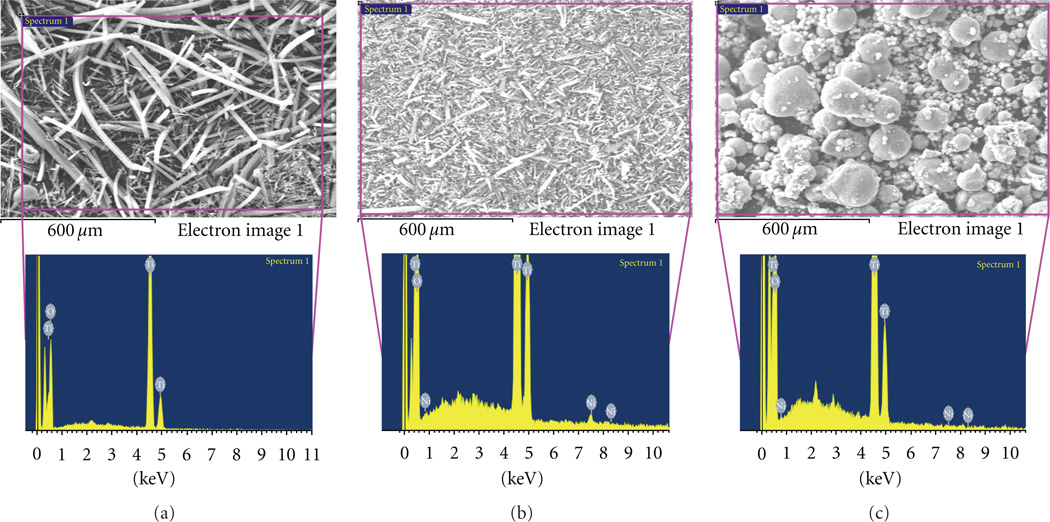
SEM images with EDX analysis. The EDX area of the pristine TiO_2_ nanofibers and its corresponding data (a), the EDX area of the modified TiO_2_ nanofibers containing Ni NPs and its corresponding data (b), the EDX area of the modified TiO_2_ microparticles containing Ni NPs and its corresponding data (c).

SEM results of the TiO_2_ microparticles containing Ni NPs are shown in (Figure 1(c)). It is observed that spherical particles are predominately present with wide range of diameters. The occurrence of particles appears in the form of clusters with the average diameters of (7000 ± 3607 nm). To verify the addition of Ni nanoparticles into the nanofibers and microparticles, SEM-EDX analysis was performed on the obtained structures (Figure 2). As shown in (Figure 2(a)) and its corresponding EDX data from the area analyses of the nanofiber, the presence of Ti from this image clearly corresponds to the occurrence of pristine TiO_2_ nanofibers. The EDX data of its counterpart which contains certain amount of Ni in the nanofibers is also present in (Figure 2(b)). From this figure we can clearly find, that in addition to the peaks of Ti, there is obvious presence of Ni peaks. This spectrum clearly demonstrates that small-sized NPs can be embedded in the nanofibres. Besides, this it can be clearly seen that diameter of these nanofibers containing Ni NPs is comparatively less than the pristine nanofibers (i.e., Figure 2(a)). This observation about the SEM-EDX results corroborates the simple SEM (Figures 1(a) and 2(b)) results, which elucidate the decrease in size of nanofibers after the addition of conductive Ni NPs. The EDX data originating from the TiO_2_ microparticles containing Ni NPs is presented in (Figure 2(c)). In this figure, the peaks corresponding to the TiO_2_ and Ni are present.

**Figure 3:**
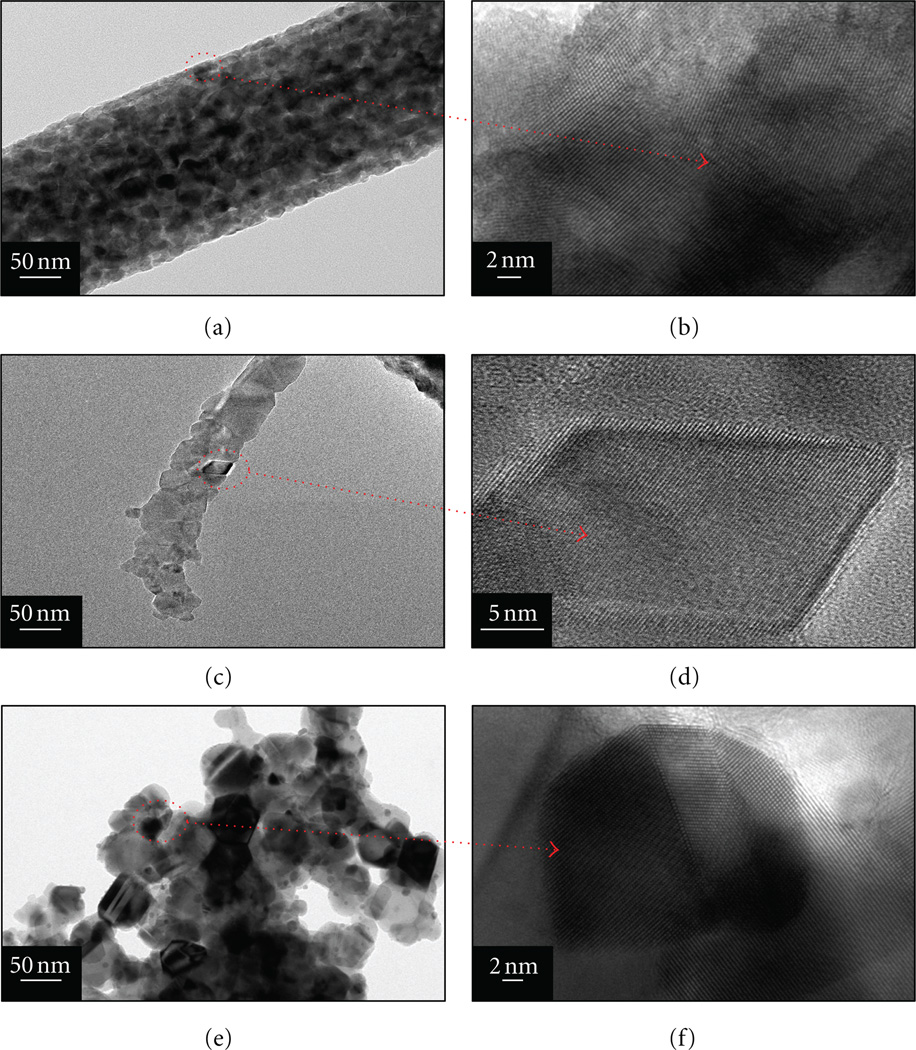
TEM images of nanofibers and microparticles after calcination process. Pristine TiO_2_ nanofibers in low magnification (a), the HRTEM images of the former figure (b). The low magnification images of the TiO_2_ nanofibers containing Ni NPs (c), the HR-TEM images of the corresponding former figure. The low magnification images of the TiO_2_ microparticles containing Ni NPs (e), the HR-TEM images of the corresponding figure (f).

It is well known, that TEM can be utilized to differentiate between two different materials in regards to their different crystalline patterns. Therefore, to investigate the crystalline features of the prepared materials, TEM images were obtained and presented in (Figure 3). Figure 3(a) shows the results from pure TiO_2_ nanofibers, where it can be seen that the morphology of individual nanofibers is consistent with that of the defect-free morphology obtained from SEM images (Figure 1(a)). The high resolution transmission electron microscope (HR-TEM) image of the pristine nanofibers is presented in (Figure 3(b)). The portion indicated by arrow in the HR-TEM image indicates that there is no dislocation of the crystal lattice and the crystal planes are arranged in a linearly unique pattern consistent of pure TiO_2_ [34]. Figure 3(c) presents TEM images of the nanofibers containing Ni NPs at low magnification, where it is confirmed that the nanoparticles are present on the nanofiber surfaces. These Ni NPs can be seen as darker areas than the main TiO_2_ nanofiber, as indicated by the arrows. HRTEM images of the marked areas in (Figure 3(c)) are presented in (Figure 3(d)). Overall, these images reveal the expected cubic shape for the Ni NPs, with diameters from 15 to 20 nm, is present. (Figure 3(e)) represents the low magnification TEM image of the microparticles containing Ni NPs. The arrow originating from encircled area shows the HR-TEM of the Ni NPs in (Figure 3(f)). It can be clearly seen, that Ni NPs are well associated with TiO_2_ microparticles. In these figures, it can be seen that crystals have good atomic arrangement with respect to two components. The atoms can be seen as uniformly arranged having regular and periodic behavior indicating individual TiO_2_ and Ni components in them, which overall indicate good crystallinity of the synthesized structures. Therefore, one can come up to an interesting finding that the crystal patterns of the both components are arranged in a leaner format and are without the dislocations or imperfections in the lattice planes. It is noteworthy to mention that in case of SEM analyses the presence of Ni NPs in case of nanofibers and microparticles was not so obvious, rationale is due high intensity electron beam of TEM which can differentiate the internal as well as external contents of the crystalline materials, of which former one is incapable to do alone, due to its poor resolution.

**Figure 4:**
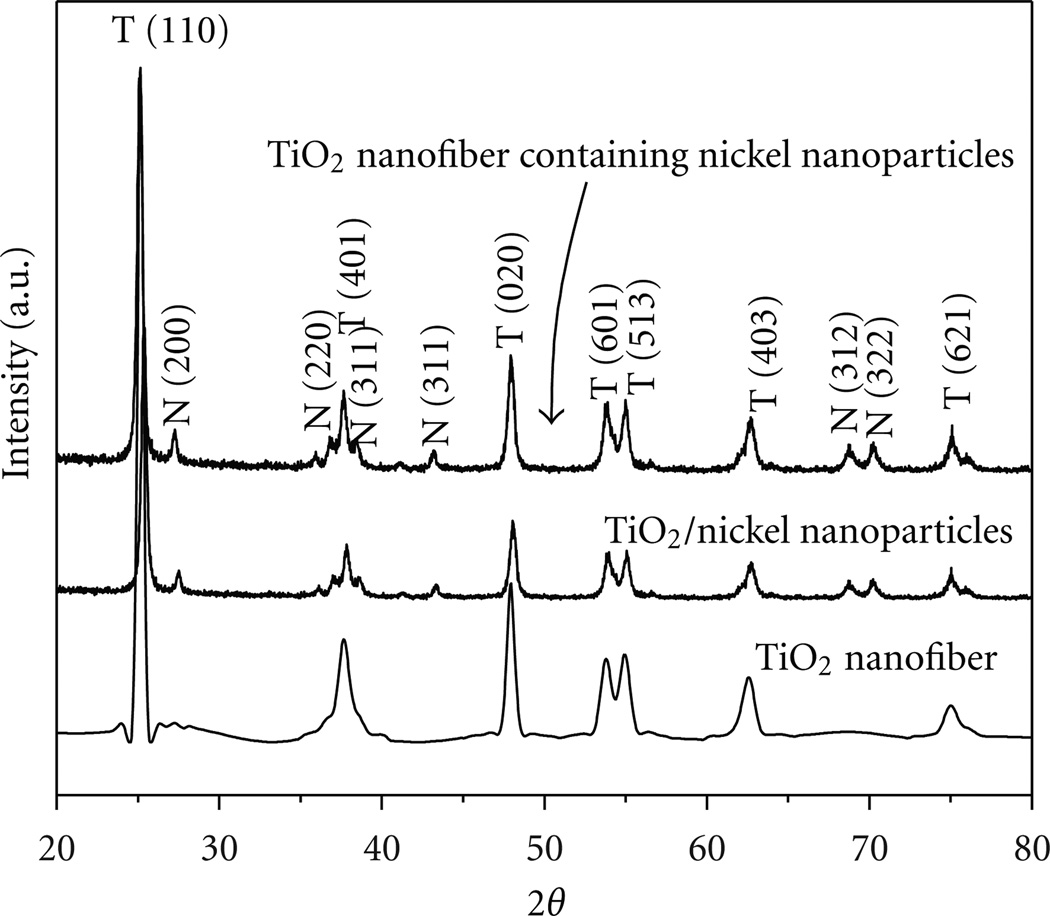
XRD results of the different nanocomposites.

As shown in (Figure 4), the spectra show the XRD pattern of pristine TiO_2_ nanofibers, TiO_2_ nanofibers containing Ni NPs, and TiO_2_ microparticles containing Ni NPs. In all of the materials obtained, there are strong diffraction peaks at 2θ values of 25.50, 37.69, 48.04, 53.85, 55.08, 62.75, and 79.98°, which correspond to the crystal planes (110), (401), (020), (601), (513), (403), and (621), respectively, and indicate the formation of pure anatase titanium dioxide [35]. Further, the TiO_2_ nanofibers containing Ni NPs and TiO_2_ microparticles containing Ni NPs presented the TiO_2_ peaks as well as the additional peaks attributed to Ni at 2θ values of 27.50, 36.80, 38.35, 43.16, 62.75, 70.33, and 79.98° corresponding to the crystal planes (220), (311), (311), (312), and (322) [36], thus confirming the presence of the embedded Ni NPs. It is interesting to note that the intensities of the Ni peaks in these composites were the same, which to some extent is expected, since the metal concentrations used were the same.

**Figure 5:**
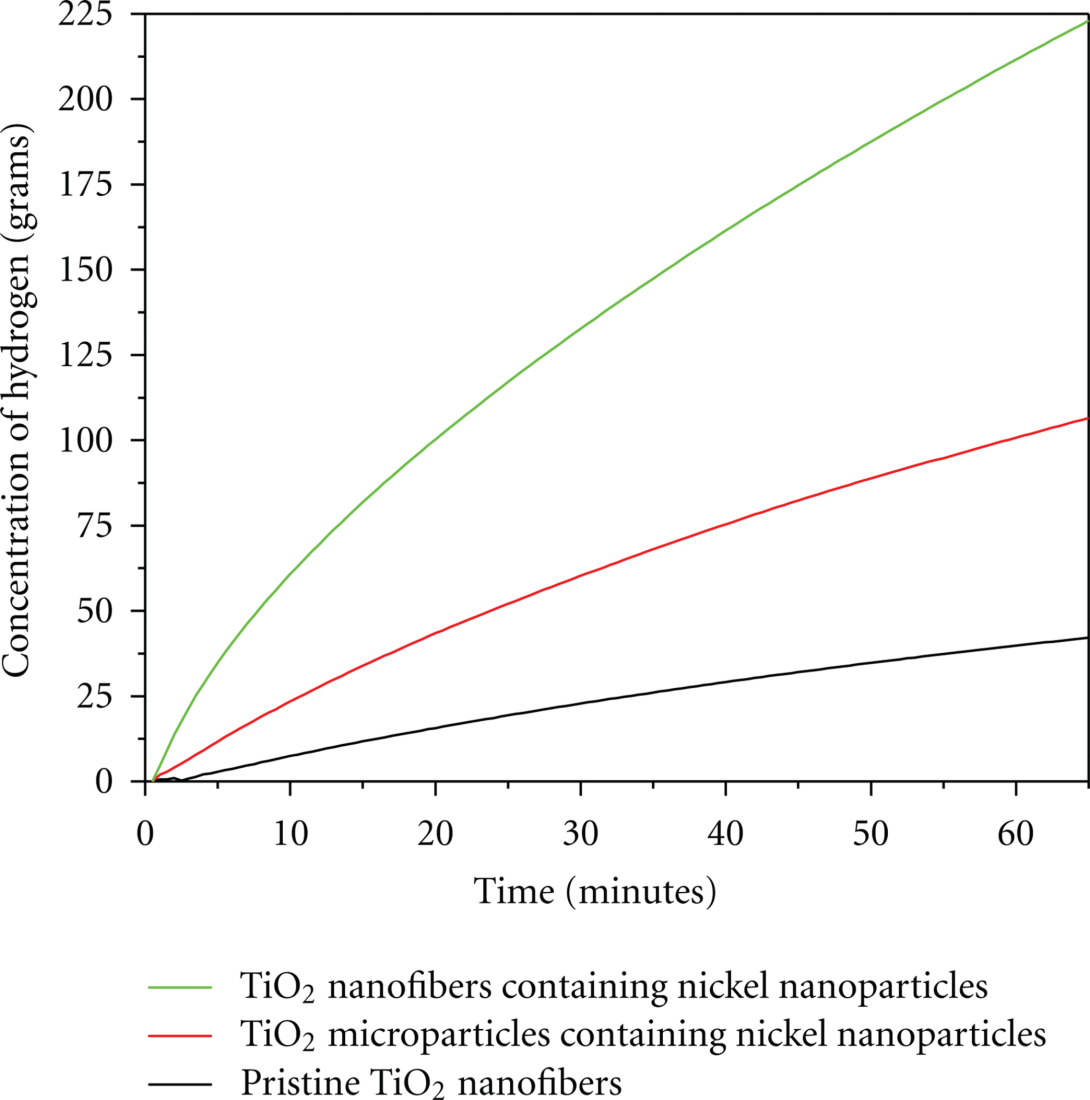
Hydrogen production of the by various nanocomposites at 26°C in pH 7.4 distilled water to demonstrate the highest hydrogen production.

Figure 5 shows the results obtained after using these synthesized structures as nanocatalysts for hydrogen production. These results demonstrate the capability of all prepared catalyst combination render to produce different amounts of hydrogen. Overall, the hydrogen production was in the range of 25 to 250 grams depending upon the nanocatalyst investigated. In more details, it is observed that the highest amount of hydrogen production is shown by the TiO_2_ nanofibers containing Ni NPs, followed by TiO_2_ microparticles containing Ni NPs, and finally by the pristine nanofibers. The main reason for the higher amount of hydrogen production from the first two nanocatalysts is clearly attributed due to the presence of Ni NPs [13]. The amount of hydrogen produced by the pristine TiO_2_ nanofibers (Ni free) is due to the presence of the original NaHB_4_ in the processing media (without catalyst).Moreover, the TiO_2_ nanofibers containing Ni NPs produce more hydrogen than its TiO_2_ microparticle containing Ni NPs analog. It is proposed that the higher surface to volume ratio present in the nanofibers is responsible for this effect [29].

To practically investigate that nanofibers do have higher surface to volume ratio than that of microparticles, which in turn can put more light, the surface area of both fibers and microparticles has been measured by using Brunauer-Emmett-Teller (BET) technique (ASAP 2010,Micromeritics, Norcross, GA). It is noteworthy to mention that exact amount of 0.5 g of Ni NP was used to mix with sol-gel to fabricate nanofibers and microparticles, therefore one would assume that two combinations would produce same results. However, from those tests, it was observed that microparticles containing Ni NPs had surface area of 13.6765 ± 0.675 m^2^/g. The pure TiO_2_ nanofibers had a surface area of 15.3456 ± 0.1751 m^2^/g, and the nanofibers containing Ni NPs had a surface area of 23.2782 ± 0.1961 m^2^/g, respectively. These results are in accordance with our previous reports [29]. Overall, these numbers indicate that nanofibers with Ni had much higher surface area, followed by pure TiO_2_ nanofibers and TiO_2_ microparticles containing Ni NPs. Furthermore, this higher surface to volume ratio in case of TiO_2_ nanofibers containing Ni NPs self explains the reason for more hydrogen production than the other two combinations.

## 4. Conclusion

In conclusion, we were able to fabricate three different types of nanomaterials through combination of sol-gel and electrospinning process. Electrospinning of a colloid comprised of Ti(Iso) and Ni NPs produced ceramic nanofibers that contained attached Ni NPs and partially captured NPs. The SEM instrument was used to find out the morphologies of nanomaterials after the electrospinning and calcinations of sol-gel. SEM equipped with EDX technique was used to differentiate between pristine and Ni-loaded nanofibers and microparticles composites. TEM images were used to determine the appearance of Ni NPs over the individual nanofibers. All the materials obtained were evaluated for their catalytic activity towards hydrogen production, resulting in the nanofibers containing Ni NPs being the most promising materials for this application was due to high surface to volume ratio of nanofibers containing Ni NPs resulted to produce the highest production of hydrogen.
